# Changes in Metabolic Health and Sedentary Behavior in Obese Children and Adolescents

**DOI:** 10.3390/jcm12175456

**Published:** 2023-08-23

**Authors:** Maciej Kochman, Marta Brzuszek, Mirosław Jabłoński

**Affiliations:** 1 Physiotherapy Department, Institute of Health Sciences, College of Medical Sciences, University of Rzeszów, Marszałkowska 24, 35-215 Rzeszów, Poland; 2 Institute of Health Sciences, College of Medical Sciences, University of Rzeszów, Kopisto 2a, 35-959 Rzeszów, Poland; 3 Chair of Rehabilitation and Physiotherapy, Faculty of Health Sciences, Medical University of Lublin, Jaczewskiego 8 Street, 20-090 Lublin, Poland

**Keywords:** obesity, metabolic disorders, cardiometabolic risk factors, children, adolescents

## Abstract

Obesity is becoming more common among children and adolescents. As in adults, obesity in the pediatric population is associated with an increased risk of metabolic disorders and diseases. In the related literature, little attention has been devoted to evaluating how metabolic health and sedentary behavior change in the obese pediatric population. Therefore, this study aimed to assess changes in metabolic health and sedentary behavior in obese children aged 7–12 and adolescents aged 13–17. For this single-center hospital-based prospective observational study, we included 202 Polish children and adolescents aged 7–17 years. We performed blood pressure measurements and collected blood samples to assess metabolic health markers. Based on the performed measurements, we also calculated additional indexes and ratios: BMI, WHtR, ABSI, VAI, and HOMA-IR. The analysis of the results showed clear and significant differences between the study groups. The older boys and girls were identified with higher values of anthropometric ratios, blood pressure, time spent sitting, and lower HDL cholesterol values (*p* < 0.05). The analysis also revealed a strong-to-moderate correlation between age and anthropometric ratios, blood pressure, HDL cholesterol, and sitting time (*p* < 0.05). Obese children and adolescents included in this study represent poor metabolic health and are at great risk of developing other metabolic diseases such as type 2 diabetes, hypertension, or metabolic syndrome. This risk increases with age; therefore, a number of preventive and therapeutic actions should be taken in overweight and obese children and adolescents to avoid further metabolic complications.

## 1. Introduction

Obesity is a chronic metabolic disease with complex interactions between genetic, environmental, and biological factors. It has been recognized as one of the diseases of affluence and is a real threat to human health worldwide [1]. The excess of adipose tissue that accumulates in the body is caused by a disturbance in the proportion between food supply and energy expenditure, resulting from the mutual and complicated interaction of environmental and genetic factors [2]. The appearance of obesity is significantly correlated with the risk of many other diseases, such as metabolic syndrome, type 2 diabetes, metabolic dysfunction-associated fatty liver disease (MAFLD), nonalcoholic steatohepatitis (NASH), selected cancers, or cardiovascular disease [3,4]. According to World Health Organization (WHO) reports, obesity is becoming more common among children and adolescents [5]. In the pediatric population, as in adults, obesity is associated with an increased risk of metabolic disorders and diseases [5,6], such as increased blood pressure and cholesterol levels [7], impaired glucose tolerance, insulin resistance, and type 2 diabetes [8], fatty liver [8,9], accelerated puberty in girls [10], or psychological and social problems [11,12,13]. In both epidemiological studies and the clinical environment, various anthropometric ratios assessing general and abdominal obesity have been proposed. The classic and most commonly used method for assessing body mass is the body mass index (BMI). Despite its simple measurement and ease of assessment, BMI is not the best and most appropriate way to assess obesity because it does not take into account individual body composition or body fat distribution [14]. For this purpose, different measurements are used, such as waist circumference and waist-to-height ratio (WHtR) [15,16], a body shape index (ABSI), and visceral adiposity index (VAI) [17,18]. There are also special methods to accurately assess the amount of body fat, such as body electrical bioimpedance; however, specific devices are needed for this purpose [19]. From a clinical point of view, it is important to diagnose abdominal obesity, because this type of obesity is strongly correlated with the occurrence of other diseases, such as hypertension, dyslipidemia, coronary artery disease [20], or type 2 diabetes [21].

The development of metabolic disorders is also contributed to by a sedentary lifestyle. Sedentary behavior leads to a reduction in the level of physical activity and, when combined with inadequate nutrition, becomes an important predictor of obesity in children. Decreasing time spent in a sitting position and undertaking regular physical activity have many beneficial effects, such as decreasing the risk of cardiovascular disease through reducing cholesterol and lipid levels [22].

As described above, impaired metabolic health increases serious complications and mortality; therefore, monitoring of metabolic health parameters should be crucial in public health policy, especially in the pediatric population. In the related literature, there are many reports concerning associations between obesity and metabolic disorders in the pediatric population; however, little attention has been devoted to evaluating how metabolic health changes in the obese pediatric population, especially in Poland. Therefore, this study aimed to assess changes in metabolic health in obese children aged 7–12 and adolescents aged 13–17. By conducting this research, we wanted to answer the following questions: (1) What are the differences between the study groups in the lipid profile, fasting glucose level, blood pressure, anthropometric measures, and sedentary behavior? (2) Which metabolic health markers are correlated with age?

## 2. Materials and Methods

### 2.1. Study Design and Ethics Approval

The protocol of this single-center hospital-based prospective observational study was approved by the Ethics Committee of the Medical University of Lublin (ref. No. KE-0254/13/2018) and the examinations were carried out in compliance with the Declaration of Helsinki. Before the start of the study, each participant and their parent or legal guardian were informed of the examination procedures. After that, each participant and their parent or legal guardian gave their written informed consent to participate in the study.

### 2.2. Participants

For this observational study, we recruited 283 obese children and adolescents aged 7–17 years living in the Subcarpathian region, Poland, who were referred to the 2nd Department of Pediatrics and Pediatric Endocrinology at the Clinical Hospital No. 2 in Rzeszow, Poland, from January 2018 to April 2019 due to diagnostic obesity. We excluded 81 people from the study due to their not meeting inclusion criteria (49 persons); refusal to participate or fear of examination (32 persons); therefore, for this study we included 202 participants. The following were the inclusion criteria: (1) obtained informed consent; (2) central obesity criteria according to IDF guidelines: waist circumference above the 90th percentile; (3) no genetic or hormonal disease leading to obesity (e.g., Prader Willi syndrome, Bardet–Biedl syndrome, Cushing syndrome, hypothyroidism); (4) no other neurological and orthopedic diseases or learning disabilities. We excluded participants who (1) did not consent to the study or were afraid of study procedures at any stage of the study, (2) with waist circumference below the s90th percentile, (3) with any genetic or hormonal disease affecting obesity, or (4) with other neurological or orthopedic diseases and learning disabilities. The study group was divided into two subgroups: the younger group aged 7–12 and the older group aged 13–17. The younger group consisted of 100 participants (32 girls and 68 boys) and the older group consisted of 102 participants (44 girls and 58 boys). The detailed characteristics of the groups are shown in Table 1.

### 2.3. Anthropometric and Metabolic Health Measures

Anthropometric measurements were performed by a qualified physiotherapist. Body weight and height were measured using a calibrated Seca scale with a height gauge. The participants were weighed in the clothes they were currently wearing during their stay at the clinic. They were weighed without shoes and outer clothing and the results were recorded to the nearest 0.1 kg. Height was measured with an accuracy of 0.1 cm [23]. Waist circumference was measured twice using a measuring tape. During the measurement, participants were asked to remain in a standing position. The waist circumference was measured in the middle of the distance between the last free rib and the upper edge of the iliac crest with an accuracy of 0.1 cm. The arithmetic mean of two measurements was included in the statistical analysis [24].

Blood samples were collected via phlebotomy from fasting patients (at least 8 h of fasting) in the morning (between 7 and 9 am) of the second day of stay at the clinic by qualified nurses using an S-Monovette blood collection system. Blood analysis was performed after centrifugation of the blood samples. The level of total cholesterol, HDL cholesterol, LDL cholesterol, and triglycerides was analyzed by Trinder reaction-based assays (Atellica CH Analyzer). The level of fasting glucose was analyzed by enzymatic hexokinase and glucose-6-phosphate dehydrogenase methods (Atellica CH Analyzer), and insulin was analyzed by chemiluminometric sandwich immunoassay (Atellica IM Analyzer). Blood analysis was performed in a clinical hospital laboratory meeting the accreditation and quality requirements for a clinical hospital.

Blood pressure was measured in the seated position by qualified nurses using a non-invasive Mindray BeneView T5 cardiomonitor according to standard blood pressure measurement procedures. This device has age-specific cuff widths dedicated for newborns, children and adults. Participants were asked to rest for 5 min before their blood pressure was measured. Three blood pressure measurements were taken and the arithmetic mean of all measurements was included in the statistical analysis [25].

Based on the performed measurements, we also calculated additional indexes and ratios: Body mass index (BMI) was calculated as BMI = weight (kg)/height (m)^2^; Waist to height ratio (WHtR) was calculated as WHtR = WC (m)/height (m); Body shape index (ABSI) was calculated as ABSI = WC (m)/(BMI^2/3^ × height (m)^1/2^); Visceral adiposity index (VAI) was calculated separately for boys and girls according to the following formulas: VAI (boys) = (WC/(1.88 × BMI + 39.68)) × (TG/1.03) × (1.31/HDL) and VAI (girls) = (WC/(1.89 × BMI + 36.58)) × (TG/0.81) × (1.52/HDL); HOMA-IR was calculated as follows: HOMA-IR = (fasting insulin (mU/mL) × fasting glucose (mg/dL))/405 [26,27,28].

Finally, we conducted a short survey on the time spent daily in a sitting position during the common school day. Questions included information about school time and homework, time devoted to computer games, watching television, as well as spontaneous and organized activities during the past week in hours and minutes.

### 2.4. Statistical Analysis

Statistical analysis was performed using Statistica 13.3 software. The Shapiro–Wilk test revealed that the investigated variables were not normally distributed; therefore, nonparametric tests were used for further statistical analysis. Statistical significance was assumed if *p* < 0.05. The Mann–Whitney U test was used to compare quantitative data corresponding to blood tests, anthropometric measures, and sitting time. The relationships between age, sitting time, and metabolic health markers were evaluated using the Spearman’s rank correlation coefficient. Statistical significance was assumed if *p* < 0.05.

A sample size calculator was used to determine the minimum sample size for the population studied. A fraction size of 0.5 was applied with a maximum error of 5%, and as a result a sample size of 173 people was obtained. Finally, 202 individuals were enrolled in the study group.

## 3. Results

The evaluation of anthropometric measurements and ratios showed significant differences in BMI, WHtR, ABSI, and VAI between gender-specific age groups. Girls aged 7–12 were identified with lower mean and median values of BMI (26.91 ± 3.44 and 25.81 vs. 31.63 ± 4.5 and 30.8) and greater mean and median values of ABSI (0.077 ± 0.003 and 0.077 vs. 0.074 ± 0.003 and 0.074) compared to girls aged 13–17. In boys aged 7–12, lower mean and median values of BMI, WHtR, VAI, and greater values of ABSI were also found in those aged 7–12 (28.6 ± 4.5 and 27.11 vs. 31.21 ± 4.6 and 30.57; 0.59 ± 0.05 and 0.6 vs. 0.57 ± 0.056 and 0.56; 3.63 ± 3.16 and 2.5 vs. 4.06 ± 2.3 and 3.15; 0.079 ± 0.004 and 0.079 vs. 0.076 ± 0.005 and 0.075, respectively). Similar results were obtained comparing age groups regardless of gender.

According to metabolic health markers, significant differences were identified between study groups in systolic and diastolic blood pressure, total cholesterol, HDL—cholesterol, LDL—cholesterol, and HOMA-IR. In girls aged 7–12, the mean and median values of systolic (114.56 ± 14.08 and 113 vs. 126.63 ± 13.93 and 128) and diastolic blood pressure (70.11 ± 7.42 and 71 vs. 76.27 ± 7.17 and 76.67) were lower and the values of total cholesterol (184.62 ± 22.56 and 184 vs. 168.93 ± 27.54 and 171) and LDL—cholesterol (117.21 ± 18.75 and 117 vs. 103.5 ± 24.91 and 105.5) were higher compared to the girls aged 13–17. Boys aged 7–12 were found with lower mean and median values of HOMA-IR (2.48 ± 1.47 and 1.9 vs. 2.76 ± 1.08 and 2.84) and systolic blood pressure (121 ± 10.74 and 120.33 vs. 130.11 ± 13.53 and 129.33) and greater values of HDL (46.4 ± 9.31 and 48 vs. 43.53 ± 7.87 and 43) and LDL—cholesterol (112.94 ± 29.96 and 112.5 vs. 102.81 ± 36.08 and 100) compared to boys aged 13–17. The analysis of age groups regardless of gender showed similar differences.

A comparison of sitting time revealed that both girls and boys aged 13–17 spent significantly more time in a sitting position than the younger group (615.95 ± 133.91 and 600 vs. 531.94 ± 154.51 and 540; 592.07 ± 124.64 and 600 vs. 532.16 ± 127.97 and 540, respectively). Detailed results are shown in Table 2.

We also evaluated the relationship between age and anthropometric measurements and ratios, as well as metabolic health markers. We observed strong-to-weak correlations between analyzed parameters. This relationship is presented separately for the gender-specific age groups and the entire population studied.

For the girls aged 7–12, a highly significant, positive, and strong-to-moderate correlation was identified between age and VAI (R = 0.61, *p* < 0.001), HOMA-IR (R = 0.56; *p* = 0.001), triglycerides (R = 0.59, *p* < 0.001), sitting time (R = 0.4, *p* = 0.025) and fasting glucose (R = 0.38, *p* = 0.031). A significant and moderate but negative correlation was identified between age and WHtR (R = −0.46, *p* = 0.011) and HDL cholesterol (R = −0.46, *p* = 0.011). In boys aged 7–12, the only correlation was found between age and HOMA-IR (R = 0.33, *p* = 0.006). For the entire group aged 7–12, a highly significant, weak, and positive correlation was observed between age and BMI (R = 0.23, *p* = 0.022), HOMA-IR (R = 0.38, *p* = 0.0001), systolic blood pressure (R = 0.29, *p* < 0.004), and sitting time (R = 0.3, *p* = 0.003). In addition, a significant, weak but negative correlation was found between age and HDL cholesterol (R = −0.28, *p* = 0.005).

For the girls aged 13–17, a significant, positive, and moderate correlation was identified between age and BMI (R = 0.35; *p* = 0.021) and a moderate but negative correlation between age and HOMA-IR (R = 0.45, *p* = 0.003) and fasting glucose (R = −0.57, *p* < 0.0001). For the boys aged 13–17, a highly significant, positive, and moderate correlation was observed between age and BMI (R = 0.39, *p* = 0.003), VAI (R = 0.42, *p* = 0.001), systolic blood pressure (R = 0.41, *p* = 0.002), triglycerides (R = 0.33, *p* = 0.012) and sitting time (R = 0.43, *p* = 0.0009). A moderate but negative correlation was found between age and ABSI (R = −0.26, *p* = 0.045), and HDL cholesterol (R = −0.38, *p* = 0.003). For the entire group aged 13–17, a highly significant, moderate, and positive correlation was identified between age and BMI (R = 0.38, *p* < 0.0001), VAI (R = 0.23, *p* = 0.02), systolic blood pressure (R = 0.26, *p* = 0.01), and sitting time (R = 0.25, *p* = 0.014). A significant, moderate but negative correlation was found between age and HDL cholesterol (R = −0.21, *p* = 0.037), fasting glucose (R = −0.2, *p* = 0.0497), and ABSI (R = −0.29, *p* = 0.003).

For the entire group of girls, a highly significant, moderate, and positive correlation was identified between age and BMI (R = 0.58, *p* < 0.0001), systolic (R = 0.37, *p* = 0.001) and diastolic blood pressure (R = 0.29, *p* = 0.011), and sitting time (R = 0.27, *p* = 0.021). A significant, strong-to-moderate, but negative correlation was found between ABSI (R = −0.61, *p* < 0.0001) and LDL cholesterol (R = −0.27, *p* = 0.022). For the entire group of boys, a highly significant, positive, and moderate correlation was found in BMI (R = 0.44, *p* < 0.0001), VAI (R = 0.26, *p* = 0.004), HOMA-IR (R = 0.29, *p* = 0.001), systolic blood pressure (R = 0.42, *p* < 0.0001) and sitting time (R = 0.36, *p* < 0.0001). A negative and moderate correlation was found between age and ABSI (R = −0.42, *p* < 0.0001) and HDL cholesterol (R = −0.3, *p* = 0.0006). As in the group of boys, similar relationships were found when we analyzed the entire studied population regardless of gender. These results are shown in Table 3.

## 4. Discussion

In this study, we evaluated how metabolic health changes between obese children aged 7–12 and adolescents aged 13–17 years. We observed significant differences between gender-specific age groups in analyzed anthropometric and metabolic health parameters. The values of BMI and blood pressure were higher in both older boys and girls, while the values of ABSI and LDL cholesterol were higher in younger boys and girls. Girls aged 7–12 were identified with greater values of total cholesterol while only boys with HDL cholesterol were identified. The comparison of metabolic parameters between the age groups regardless of gender revealed similar results, as the older group was identified with greater values of BMI, blood pressure, WHtR, and VAI, while the values of ABSI, HDL, and LDL cholesterol and total cholesterol were higher in the younger group. We did not observe differences in levels of triglycerides and fasting glucose between groups. As is commonly known, the human body changes with age, especially during puberty. In this period, there are dynamic changes in the external appearance of boys and girls, and the whole body and its proportions change due to growth and weight gain [29]. However, higher values of anthropometric parameters and lower values of HDL cholesterol in the older group are a cause for concern, as in the literature there is strong evidence of associations of these factors with diabetic complications [30], cardiovascular [31], and cardiometabolic risks [32]. Moreover, HDL cholesterol plays a critical protective and antiatherogenic role [33]. We also observed higher values of total cholesterol and LDL cholesterol in the younger group. These parameters are also a cause for concern, as they correlate with hypertension [34] and atherosclerotic cardiovascular disease [35].

The above results are also consistent with the correlations we performed between age and anthropometric ratios, as well as metabolic health markers. Our results showed strong-to-weak positive correlations between age and BMI, HOMA-IR, VAI, and systolic blood pressure. The negative strong-to-weak correlations were noticed between age and HDL cholesterol level and ABSI. These results indicate that the metabolic health of the study group may deteriorate in the future unless preventive actions are taken to stop or reverse this process [36,37]. In some studies, authors observed a relationship between the stage of puberty and the development of metabolic disorders. According to Boyne et al., an earlier onset of puberty is associated with increased cardiometabolic risk only in girls. Their study showed that earlier menarche and greater breast development in girls were related to higher fasting glucose and the pubarchal stage was associated with systolic blood pressure [38].

Sedentary behavior, as well as insufficient physical activity, passive leisure activities, high-calorie diets, and unhealthy eating habits, may lead to metabolic disorders and various health problems [39,40,41]. In this study, the comparison of sitting time revealed that both girls and boys aged 13–17 spent significantly more time in a sitting position than the younger boys and girls. We also observed a positive and moderate correlation between age and sitting time in almost every analyzed group. This has been also observed in a longitudinal study by Janssen et al. of English children and adolescents [42]. According to Martinez- Gomez et al., the time spent in sedentary activities can play a critical role in the development of cardiovascular risk during adolescence. The results of their study suggest the need to take into account a reduction in sedentary behavior as an additional preventive strategy for the premature development of cardiovascular risk in infancy and adolescence, as well as the promotion of physical activity and improvements in eating habits [43]. The study results of Govindan et al. indicate that increased time spent watching television was significantly related to obesity for both boys and girls aged 10–12 and this relationship was influenced by corresponding decreased physical activity. The authors suggest that interventions reducing sedentary behaviors may be an important component of reducing childhood obesity [44]. This has also been confirmed in other studies [45,46]. The cohort study carried out by Aljahdali et al. showed that sedentary time was related to cardiometabolic risk factors in Mexican children and adolescents. The authors indicated that replacing sedentary time with higher intensities improves cardiometabolic markers [45]. The study conducted by Velde et al. revealed that sedentary time was positively associated with BMI z-score and waist circumference in Dutch primary school children aged 7–12 years [46].

The results of this research are alarming and should be taken into account by governments and national health systems to promote preventive, educational, and therapeutic actions and health programs, especially in overweight and obese children and adolescents, and their parents. This study may also have clinical applications for primary care physicians and other clinical specialists during routine health check-ups in children and adolescents. Finally, as the study participants were primary and high school students, this study may have an educational impact on schoolteachers and principals to better understand the health-related problems of students.

We must admit that this study has some limitations. The primary limitation of our study is that the study groups were unequal. In both age groups, there were fewer girls and that could disturb the comparison between groups. Because this was a hospital-based study, we were also unable to include a control group to prove whether changes in metabolic health were related to the obesity or age-related growth of children. Finally, it could be beneficial to include physical activity and nutritional assessments to further investigate the metabolic health status of study participants.

## 5. Conclusions

In our study, we observed critical changes between gender-specific age groups in anthropometric and metabolic health parameters. The comparison of metabolic parameters between the age groups regardless of gender revealed that the older group was identified with greater values of BMI, blood pressure, WHtR, and VAI, while the values of ABSI, HDL and LDL cholesterol, and total cholesterol were higher in the younger group. The comparison of gender-specific age groups indicated that the values of BMI and blood pressure were higher in both older boys and girls, while the values of ABSI and LDL cholesterol were higher in younger boys and girls. Girls aged 7–12 were identified with greater values of total cholesterol while boys were identified with HDL cholesterol. We also demonstrated strong-to-weak positive correlations between age and BMI, HOMA-IR, VAI, and systolic blood pressure, but also negative strong-to-weak correlations between age and HDL cholesterol level and ABSI. We also observed that sedentary behavior worsens with age in both boys and girls. The correlation between age and sitting time was positive and moderate in almost every analyzed group. Obese children and adolescents are at great risk of developing further metabolic diseases such as type 2 diabetes, hypertension, or metabolic syndrome. The risk increases with age; therefore, a number of preventive and therapeutic actions should be taken in overweight and obese children and adolescents to avoid further metabolic complications. They should also be encouraged to follow a healthy lifestyle, including a healthy and low-calorie diet, and engage in various forms of physical activity to reduce sitting time and ultimately improve their metabolic health status. Because obesity is now considered a worldwide epidemic increasing comorbidities’ prevalence and metabolic disorders, it is important to monitor the metabolic health parameters, especially in the pediatric population. Further studies including control groups, physical activity and nutritional assessments are needed in this area.

## Figures and Tables

**Table 1:** Characteristics of the study group.

Variable		Group Aged 7–12 (n = 100)		Group Aged 13–17 (n = 102)		Total (n = 202)	
		Mean	SD	Mean	SD	Mean	SD
Age	Girls	10	1.67	14.82	1.42	12.79	2.83
	Boys	10.57	1.15	14.53	1.35	12.4	2.34
	Total	10.39	1.36	14.66	1.38	12.55	2.54
Body mass [kg]	Girls	61.09	14.76	84.7	12.97	74.76	18.01
	Boys	69.36	18.7	93.41	20.11	80.43	22.73
	Total	66.72	17.89	89.65	17.85	78.3	21.21
Height [m]	Girls	1.5	0.12	1.64	0.07	1.58	0.12
	Boys	1.54	0.11	1.72	0.1	1.63	0.14
	Total	1.53	0.12	1.68	0.1	1.61	0.13

**Table 2:** Comparison of anthropometric measurements, metabolic health markers, and sitting time between groups.

Variable	Gender	Group Aged 7–12 (N = 100)		Group Aged 13–17 (N = 102)		Z	*p*
		Mean (SD)	Median	Mean (SD)	Median		
BMI	Girls	26.91 (3.44)	25.81	31.63 (4.5)	30.8	−4.68	<0.0001
	Boys	28.6 (4.5)	27.11	31.21 (4.6)	30.57	−3.81	<0.0001
	Total	28.06 (4.25)	27	31.39(4.54)	30.78	−5.81	<0.0001
WHtR	Girls	0.57 (0.05)	0.55	0.58 (0.06)	0.57	−0.43	0.668
	Boys	0.59 (0.05)	0.6	0.57 (0.056)	0.56	2.73	0.006
	Total	0.59 (0.05)	0.58	0.57 (0.06)	0.56	1.93	0.05
ABSI	Girls	0.077 (0.003)	0.077	0.074 (0.003)	0.074	5.05	<0.0001
	Boys	0.079 (0.004)	0.079	0.076 (0.005)	0.075	4.52	<0.0001
	Total	0.079 (0.003)	0.077	0.075 (0.004)	0.074	6.73	<0.0001
VAI	Girls	4.54 (2.45)	3.94	4.78 (3.16)	3.98	−0.05	0.96
	Boys	3.63 (3.16)	2.5	4.06 (2.3)	3.15	−2.29	0.02
	Total	3.89 (2.99)	3.28	4.36 (2.7)	3.28	−2.14	0.03
HOMA-IR	Girls	3.48 (2.44)	2.74	3.33 (2.42)	2.48	0.55	0.58
	Boys	2.48 (1.47)	1.9	2.76 (1.08)	2.84	−1.89	0.06
	Total	2.8 (1.88)	2.11	3.01 (1.81)	2.69	−1.37	0.172
SBP	Girls	114.56 (14.08)	113	126.63 (13.93)	128	−3.31	0.001
	Boys	121 (10.74)	120.33	130.11 (13.53)	129.33	−3.65	0.0002
	Total	118.97 (12.2)	118.67	128.57 (13.74)	128.33	−4.71	<0.0001
DBP	Girls	70.11 (7.42)	71	76.27 (7.17)	76.67	−3.31	0.0009
	Boys	75.58 (5.95)	75	77.79 (9.95)	78	−1.3	0.192
	Total	73.85 (6.9)	74.67	77.12 (8.82)	77	−2.62	0.009
CL [mg/dL]	Girls	184.62 (22.56)	184	168.93 (27.54)	171	2.06	0.04
	Boys	182.12 (36.89)	181.5	173.72 (39.9)	168	1.72	0.086
	Total	182.87 (33.16)	183	171.71 (3516)	171	2.62	0.009
HDL-C [mg/dL]	Girls	46.28 (9.22)	47	43.83 (8.46)	42	1.2	0.228
	Boys	46.4 (9.31)	48	43.53 (7.87)	43	1.96	0.05
	Total	46.36 (9.24)	47	43.66 (8.08)	42	2.47	0.013
LDL-C [mg/dL]	Girls	117.21 (18.75)	117	103.5 (24.91)	105.5	2.67	0.008
	Boys	112.94 (29.96)	112.5	102.81 (36.08)	100	2.34	0.019
	Total	114.22 (27.07)	116	103.1 (31.73)	100.5	3.28	0.001
TG [mg/dL]	Girls	105.59 (43.75)	102	108 (60.58)	90.5	0.37	0.708
	Boys	120.99 (72.66)	102.5	130.69 (57)	114	−1.84	0.066
	Total	116.38 (65.53)	102	121.16 (59.31)	107.5	−1.01	0.31
FG [mg/dL]	Girls	83.56 (6.03)	84.5	82.21 (10.73)	81	0.8	0.424
	Boys	84.01 (9.76)	83	85.86 (11.9)	85	−0.77	0.439
	Total	83.87 (8.71)	83	84.29 (11.5)	83	−0.1	0.921
ST [min]	Girls	531.94 (154.51)	540	615.95 (133.91)	600	−1.99	0.046
	Boys	532.16 (127.97)	540	592.07 (124.64)	600	−2.48	0.013
	Total	532.09 (136.11)	540	602.1 (128.49)	600	−3.32	0.001

N—number of participants; Z–z-score; BMI—body mass index; WC–waist circumference; WHtR—waist to height ratio; ABSI–a body shape index; VAI—visceral adiposity index; HOMA-IR—homeostatic model assessment of insulin resistance; SBP—systolic blood pressure; DBP—diastolic blood pressure; CL—total cholesterol; HDL-C—HDL cholesterol; LDL-C—LDL cholesterol; TG—triglycerides; FG—fasting glucose; ST—sitting time.

**Table 3:** Correlation of age vs. anthropometric measurements, ratios, metabolic health markers, and sitting time.

		Aged 7–12 (N = 100)		Aged 13–17 (N = 102)		Total (N = 202)	
		R	*p*	R	*p*	R	*p*
BMI	Girls	0.21	0.251	0.35	0.021	0.58	<0.0001
	Boys	0.23	0.057	0.39	0.003	0.44	<0.0001
	Total	0.23	0.022	0.38	<0.0001	0.49	<0.0001
WHtR	Girls	−0.46	0.011	0.21	0.18	0.06	0.611
	Boys	0.16	0.203	0.01	0.951	−0.17	0.0612
	Total	−0.01	0.948	0.1	0.314	−0.09	0.206
ABSI	Girls	0.01	0.988	−0.29	0.062	−0.61	<0.0001
	Boys	−0.09	0.469	−0.26	0.045	−0.42	<0.0001
	Total	−0.01	0.91	−0.29	0.003	−0.49	<0.0001
VAI	Girls	0.61	0.0007	−0.06	0.706	0.07	0.581
	Boys	0.04	0.772	0.42	0.001	0.26	0.004
	Total	0.16	0.122	0.23	0.02	0.22	0.002
HOMA-IR	Girls	0.56	0.001	−0.45	0.003	−0.1	0.409
	Boys	0.33	0.006	0.16	0.259	0.29	0.001
	Total	0.38	0.0001	−0.15	0.151	0.16	0.027
SBP	Girls	0.27	0.137	0.1	0.54	0.37	0.001
	Boys	0.24	0.053	0.41	0.002	0.42	<0.0001
	Total	0.29	0.004	0.26	0.01	0.4	<0.0001
DBP	Girls	0.01	0.983	−0.1	0.536	0.29	0.011
	Boys	0.02	0.844	−0.05	0.722	0.08	0.41
	Total	0.1	0.33	−0.1	0.322	0.15	0.035
CL	Girls	0.08	0.675	−0.03	0.873	−0.21	0.078
	Boys	−0.03	0.825	0.223	0.093	−0.1	0.247
	Total	0.01	0.946	0.11	0.264	−0.14	0.055
HDL-C	Girls	−0.46	0.011	−0.01	0.956	−0.2	0.092
	Boys	−0.21	0.086	−0.38	0.003	−0.3	0.0006
	Total	−0.28	0.005	−0.21	0.037	−0.28	<0.0001
LDL-C	Girls	−0.09	0.646	0.05	0.74	−0.27	0.022
	Boys	−0.01	0.988	0.26	0.051	−0.14	0.131
	Total	−0.02	0.859	0.16	0.104	−0.17	0.015
TG	Girls	0.59	0.0007	−0.06	0.703	0.03	0.785
	Boys	−0.04	0.759	0.33	0.012	0.19	0.03
	Total	0.19	0.06	0.11	0.293	0.13	0.06
FG	Girls	0.38	0.031	−0.57	<0.0001	−0.23	0.051
	Boys	0.01	0.956	0.14	0.286	0.09	0.307
	Total	0.13	0.204	−0.2	0.0497	−0.01	0.845
ST	Girls	0.4	0.025	−0.01	0.971	0.27	0.021
	Boys	0.23	0.059	0.43	0.0009	0.36	<0.0001
	Total	0.3	0.003	0.25	0.014	0.34	<0.0001

N—number of participants; R—Spearman correlation coefficient; BMI—body mass index; WC—waist circumference; WHtR—waist to height ratio; ABSI—a body shape index; VAI—visceral adiposity index; HOMA-IR—homeostatic model assessment of insulin resistance; SBP—systolic blood pressure; DBP—diastolic blood pressure; CL—total cholesterol; HDL-C—HDL cholesterol; LDL-C—LDL cholesterol; TG—triglycerides; FG—fasting glucose; ST—sitting time.

## Data Availability

The datasets used during the current study are available from the corresponding author on reasonable request.
